# Supplementary material for: The Combination of Intestinal Alkaline Phosphatase Treatment with Moderate Physical Activity Alleviates the Severity of Experimental Colitis in Obese Mice via Modulation of Gut Microbiota, Attenuation of Proinflammatory Cytokines, Oxidative Stress Biomarkers and DNA Oxidative Damage in Colonic Mucosa
Source: Int J Mol Sci. 2022 Mar 9;23(6):2964. doi: 10.3390/ijms23062964 (PMC8955215; doi:10.3390/ijms23062964)
Supplement: Supplementary file 1 [file ijms-23-02964-s001.zip › ijms-1608534-supplementary.pdf]

Supplementary section material

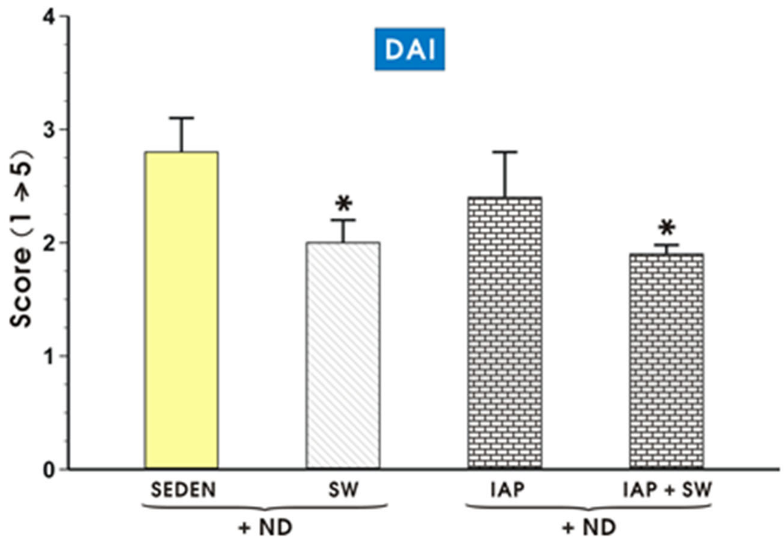

Figure S1. The effect of exercise on spinning wheels (SW) and intestinal alkaline phosphatase (IAP) administration both applied alone or in the combination SW+ IAP on the disease activity index (DAI) in normal diet fed mice (ND fed) with TNBS-induced colitis as compared to sedentary (SEDEN) mice with TNBS colitis. Results are mean ± S.E.M. of 8 animals per each group. An asterisk indicates a significant change ($p<0.05$) as compared to the respective value in the SEDEN mice with colitis.

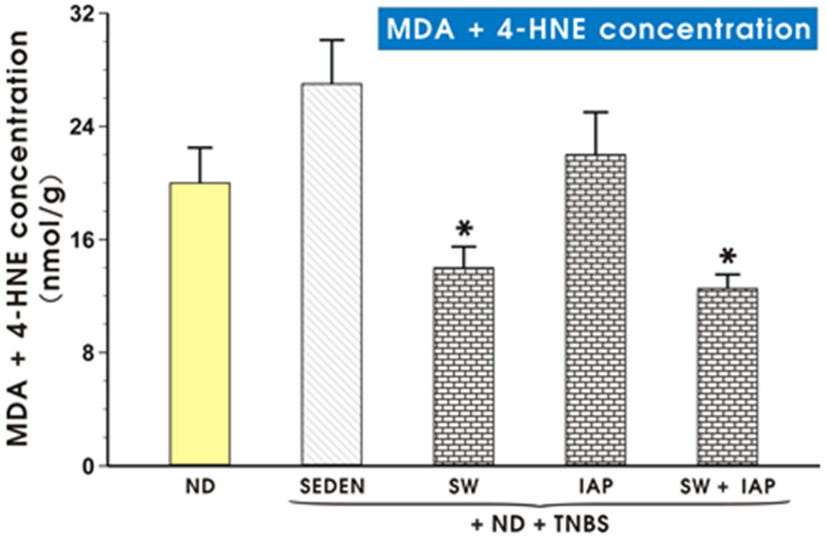

Figure S2. Lipid peroxidation products expressed as malondialdehyde and 4-hydroxynonenal (MDA + 4-HNE) concentration in colonic mucosa of a normal diet (ND) voluntary exercised TNBS colitis mice exercising on spinning wheels (SW) with or without the intestinal phosphatase (IAP) treatment or in those subjected to the combination of SW and IAP. Results are mean ± S.E.M. of 6-8 animals per each group. An asterisk indicates a significant change ($p<0.05$). as compared to the respective values in the sedentary mice (SEDEN) fed ND.

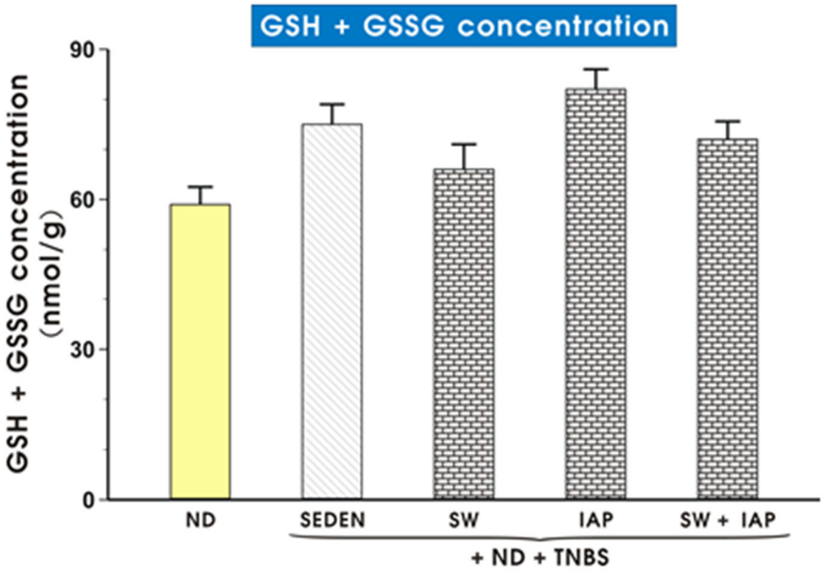

Figure S3. Total glutathione (reduced GSH+ oxidized GSSG) concentration in colonic mucosa in mice with or without TNBS colitis fed a normal diet (ND) with access to spinning wheels (SW), administered with or without the combination with the intestinal alkaline phosphatase (IAP). Results are mean ± S.E.M. of 6-8 animals per each group. The treatment with IAP tended to increase the glutathione content in colonic mucosa but this change was not significant as compared with the respective value in sedentary (SEDEN) mice with TNBS colitis.

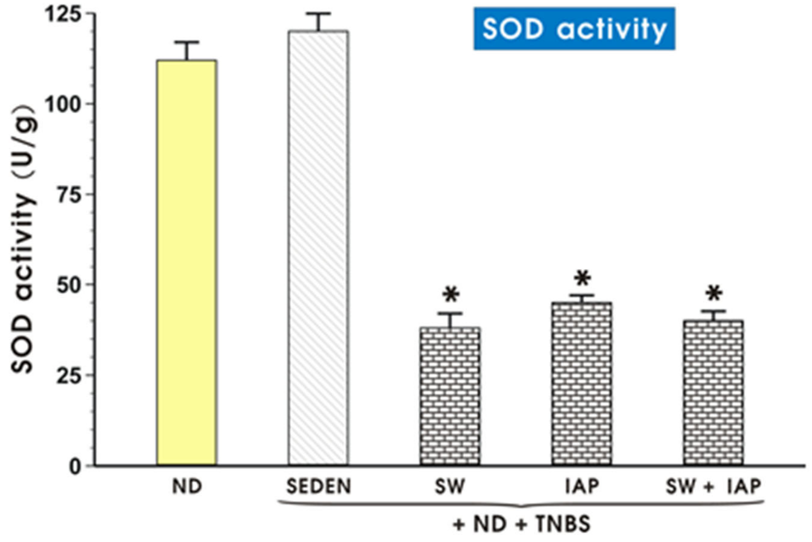

Figure S4. Determination superoxide dismutase (SOD) activity in colonic mucosa of a normal diet (ND) fed mice with or without TNBS colitis with access to spinning wheels (SW) with or without the intestinal alkaline phosphatase (IAP) treatment. Results are mean ± S.E.M. of 6-8 animals per each group. An asterisk indicates a significant change ($p<0.05$) as compared to the respective values in the sedentary (SEDEN) ND fed mice.
